# PlantNh-Kcr: a deep learning model for predicting non-histone crotonylation sites in plants

**DOI:** 10.1186/s13007-024-01157-8

**Published:** 2024-02-15

**Authors:** Yanming Jiang, Renxiang Yan, Xiaofeng Wang

**Affiliations:** 1 College of Mathematics and Computer Sciences, Shanxi Normal University, Taiyuan, 030031 China; 2 The Key Laboratory of Marine Enzyme Engineering of Fujian Province, Fuzhou University, Fuzhou, 350002 China; 3 College of Biological Science and Engineering, Fuzhou University, Fuzhou, 350002 China

**Keywords:** Crotonylation, Convolutional neural network, Bidirectional long short-term memory, Attention mechanism, Focal loss

## Abstract

**Background:**

Lysine crotonylation (Kcr) is a crucial protein post-translational modification found in histone and non-histone proteins. It plays a pivotal role in regulating diverse biological processes in both animals and plants, including gene transcription and replication, cell metabolism and differentiation, as well as photosynthesis. Despite the significance of Kcr, detection of Kcr sites through biological experiments is often time-consuming, expensive, and only a fraction of crotonylated peptides can be identified. This reality highlights the need for efficient and rapid prediction of Kcr sites through computational methods. Currently, several machine learning models exist for predicting Kcr sites in humans, yet models tailored for plants are rare. Furthermore, no downloadable Kcr site predictors or datasets have been developed specifically for plants. To address this gap, it is imperative to integrate existing Kcr sites detected in plant experiments and establish a dedicated computational model for plants.

**Results:**

Most plant Kcr sites are located on non-histones. In this study, we collected non-histone Kcr sites from five plants, including wheat, tabacum, rice, peanut, and papaya. We then conducted a comprehensive analysis of the amino acid distribution surrounding these sites. To develop a predictive model for plant non-histone Kcr sites, we combined a convolutional neural network (CNN), a bidirectional long short-term memory network (BiLSTM), and attention mechanism to build a deep learning model called PlantNh-Kcr. On both five-fold cross-validation and independent tests, PlantNh-Kcr outperformed multiple conventional machine learning models and other deep learning models. Furthermore, we conducted an analysis of species-specific effect on the PlantNh-Kcr model and found that a general model trained using data from multiple species outperforms species-specific models.

**Conclusion:**

PlantNh-Kcr represents a valuable tool for predicting plant non-histone Kcr sites. We expect that this model will aid in addressing key challenges and tasks in the study of plant crotonylation sites.

**Supplementary Information:**

The online version contains supplementary material available at 10.1186/s13007-024-01157-8.

## Introduction

Post-translational modifications (PTMs) [1] of proteins involve the addition or removal of chemical groups to amino acid residues, thereby modifying protein properties and expanding functional diversity. PTMs play crucial roles in various biological processes and metabolic pathways. Among the PTMs, lysine crotonylation (Kcr) is a novel and significant modification that has been widely detected in both animals and plants. The Kcr modification was initially discovered in the histones of human somatic cells and mouse germ cells, and Kcr enrichment on sex chromosomes has been identified as a key indicator in male germ cell differentiation control [2]. In mice, it was shown that increased histone crotonylation level might have a positive effect on acute kidney injury [3]. In addition, histone Kcr sites are involved in many other biological processes, including organism development [2], DNA damage response [4], and gene transcription and expression [5]. Kcr is not limited to histones but is also abundant in non-histones [6–9]. Previous studies have clearly revealed that crotonylation of non-histone proteins is associated with various metabolic pathways and participates in protein expression and multiple cell signaling cascades [8].

In plants, global identification and functional analysis of lysine crotonylation have been conducted in species such as tabacum [10], papaya [11], rice [12], peanut [13], and wheat [14, 15]. The Kcr modification is involved in regulating various metabolic pathways in plants, including photosynthesis, oxidative phosphorylation, and carbon metabolism [14]. Additionally, Kcr is involved in gene transcription regulation [12] and adaptation to adverse conditions in plants [16]. Notably, Kcr is related to cold stress tolerance in plants [17], exhibiting a positive regulatory effect on wheat's freezing tolerance [14].

The current experimental methods for detecting Kcr sites include high-performance liquid chromatography fractionation, stable isotope labelling of amino acids in cell culture, immunological affinity enrichment, and high-resolution liquid chromatography-tandem mass spectrometry [18]. While biological experiments are the most reliable means to identify Kcr sites, the experiments are often time-consuming, labor-intensive, and costly. In addition, mass spectrometry platforms can only identify a subset of crotonylated peptides due to factors such as protein abundance, protein hydrolysis and digestion [19]. Therefore, computational models for conveniently and rapidly predicting Kcr sites are desirable, which have been developed in the past few years.

Early models for Kcr site prediction were limited by the small training datasets with fewer than 200 Kcr sites, and all sites were limited to histones. These models employed conventional machine learning methods such as support vector machines [20], random forest (RF) [21], LightGBM [22–24], etc. The input features used by these models include composition of amino acids and amino acid pairs, amino acid properties, etc. The representative models include CrotPred [25], CKSAAP_CrotSite [26], iKcr-PseEns [27], iCrotoK-PseAAC [28], LightGBM-CroSite [29], etc.

With the advancement of mass spectrometry technology, the global Kcr sites in the proteome of several species have been detected that enabled the utilization of significantly larger training datasets. At the same time, the deep learning frameworks [30] have reached a level of maturity that resulted in the common employment of deep learning methods in establishing predictive models for Kcr sites. Among these models, some predict Kcr sites on a mixture of histones and non-histones, such as Deep-Kcr [31], BERT-Kcr [32], DeepCap-Kcr [33], Adapt-Kcr [34], and ATCLSTM-Kcr [19]. Others are tailored to predict Kcr sites on non-histones, such as nhKcr [35], DeepKcrot [36], iKcr_CNN [37], and CapsNh-Kcr [38]. The primary input features of these models consist of binary encoding and embedding vectors. A majority of these models utilize convolutional neural networks (CNNs) [19, 31, 33–38] and long short-term memory networks (LSTM) [19, 32–34, 36] as integral components of their structure. Notably, some models integrate self-attention mechanism to enhance their predictive capabilities [19, 32, 34]. Additionally, a few models stand out due to their unique network architecture. For instance, DeepCap-Kcr and CapsNh-Kcr employ capsule networks, a distinctive approach in deep learning architecture. The deep learning models have resulted in significantly improved performance compared to the earlier conventional machine learning models.

Among the four models for predicting Kcr sites on non-histones, nhKcr, iKcr_CNN, and CapsNh-Kcr are limited to predicting human Kcr sites. In contrast, DeepKcrot predict Kcr sites in four species, including humans, rice, tabacum, and papaya. However, it is important to note that due to the current unavailability of DeepKcrot's web server, accessing its datasets and models for further research purposes can be challenging. In addition, recent experimental studies have detected Kcr sites in some other plants, emphasizing the need for a computational model that is specifically tailored for plants. To address this gap, it is essential to integrate existing Kcr site data detected from plants and establish a specialized computational model dedicated to plants.

In this study, we compiled a comprehensive dataset of non-histone Kcr sites from five plant species including rice, tabacum, papaya, peanut, and wheat, and built a reliable training and test dataset. Then we utilized the binary encoding (BE) as input features and employed a combination of a convolutional neural network (CNN) [39], a bidirectional long short-term memory (BiLSTM) network [40] and multi-head self-attention (MHSA) [41] to construct a novel deep learning model called PlantNh-Kcr. This model was specifically designed to predict Kcr sites on non-histones in plants. We validated our model through rigorous comparisons with conventional machine learning methods and other state-of-the-art deep learning models. On five-fold cross-validation and independent test, PlantNh-Kcr consistently demonstrated superior performance. Furthermore, it excelled in predicting Kcr sites across individual plant species, highlighting its remarkable versatility and generalizability. We believe that the development of this plant-specific prediction model offers valuable insights for the biological community and will drive further advancement in plant biology.

## Materials and methods

### Benchmark dataset

**Fig. 1:**
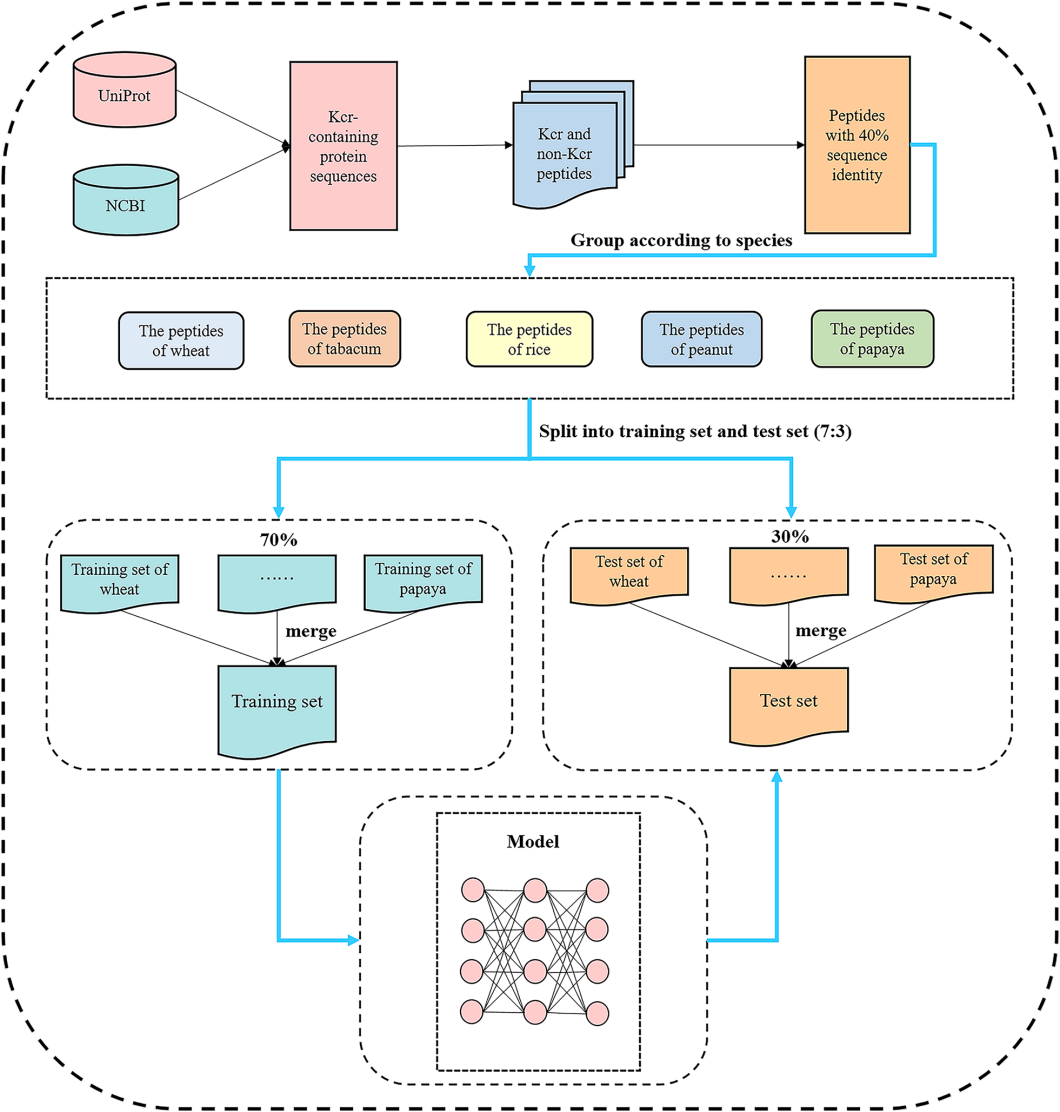
The flowchart of dataset preparation

To train and test the model, we carefully curated a training dataset and a test dataset. This process was meticulously designed and is depicted in Fig. 1. We first collected non-histone Kcr sites from five plant species. These sites numbered 5692 from wheat [14, 15], 1258 from rice [12], 2028 from tabacum [10], 6603 from peanut [13], and 5332 from papaya [11]. We then retrieved the corresponding protein sequences from the UniProt [42] and NCBI [43] databases for each species. Subsequently, we extracted peptides of length 29 from these protein sequences, with the K (Lysine) residue positioned at the center and 14 residues upstream and downstream respectively. If a peptide had fewer than 14 residues on one side, we replaced the missing residues with X. Peptides where the central K residues represented Kcr sites were designated as positive samples; otherwise, they were designated as negative samples. To eliminate redundancy and potential false negatives, we used the CD-HIT program [44] with a sequence identity threshold of 40%. Finally, we obtained 12,352 positive and 46,389 negative samples. To evaluate the performance of the model on test samples of each species, we separated the samples based on their species. For each species, the samples were randomly divided into two subsets in a 7:3 ratio while retaining the proportion of positive and negative samples during the partitioning process. The larger sets for each species were merged to form the training dataset and the smaller sets for each species were merged to form the test dataset. The training dataset totaled 41,114 samples, with 8644 positive and 32,470 negative samples. The test dataset included 17,627 samples, with 3708 positive 13,919 negative samples. The numbers of samples for each species in the training and test datasets are listed in Table 1.

**Table 1:** The numbers of positive and negative samples for each species in the training and test datasets

Species	Training set		Test set	
	Positive	Negative	Positive	Negative
Wheat	2484	7585	1066	3252
Tabacum	820	2524	352	1082
Rice	662	3486	284	1495
Peanut	2452	10,675	1051	4575
Papaya	2226	8200	955	3515
Total samples	8644	32,470	3708	13,919

### Peptide encoding

To build a predictive model for non-histone Kcr sites, it is necessary to transform peptide samples into numerical vectors as input features for the model. PlantNh-Kcr employs binary encoding as its input features. We conducted a comparative analysis of PlantNh-Kcr’s predictive performance against conventional machine learning models and other deep learning models. The conventional machine learning models utilize various input features, including amino acid composition (AAC), enhanced group amino acid composition (EGAAC), BE, AAindex encoding, and BLOSUM62 encoding. Other deep learning models employ features including BE, word embedding (WE) encoding, AAindex encoding, and BLOSUM62 encoding. The following provides a detailed description of these encoding methods:

AAC: In bioinformatics, AAC is a commonly used encoding method, which calculates the frequencies of each amino acid in a peptide. In this study, X is also considered as an amino acid. So the peptide is encoded as a 21-dimensional vector, where each dimension corresponds to the frequency of one of the 21 amino acids present in the peptide.

EGAAC: The EGAAC encoding divides amino acids into five groups based on their physicochemical properties, i.e. aliphatic group (GAVLMI), aromatic group (FYW), positively charged group (KRH), negatively charged group (DE), and no charge group (STCPNQ). A peptide is encoded as a five-dimensional vector, where each dimension represents the proportion of one of the five groups of amino acids within the peptide.

BE: BE is a common technique used to convert amino acid sequences into numerical representations suitable for model training. For this encoding method, each amino acid is encoded as a 21-dimensional binary vector. This vector has one component set to 1 to indicate the type of amino acid, while all other components are set to 0. Finally, a peptide of length 29, is encoded as a matrix or a vector of size 29 $\times$ 21.

WE encoding: WE is a technique that has gained popularity in the field of natural language processing. It assigns words to vectors in a high-dimensional space, ensuring that semantically similar words are positioned close to each other. This technique has also been effectively applied to sequence encoding in bioinformatics [45, 46]. In this work, the vocabulary size is set to 21, representing the number of amino acid types. The peptide of length 29 is treated as a sentence, with each amino acid residue mapped to a unique word ID. Subsequently, WE is used to translate these IDs into vectors. Finally, the peptide is encoded as a matrix of size 29 $\times$ 10, where 10 is the dimension of the vector space.

AAindex encoding: AAindex [47] is a public database that curates a range of physicochemical and biochemical properties of amino acids. This database serves as a valuable resource for various bioinformatics studies, including protein structure prediction, sequence alignment, protein function annotation, and more. Previously, the model nhKcr selected 29 indices from AAindex that were most relevant to the prediction task to encode the peptide [35]. In this study, we used the same 29 indices to encode the peptide. Consequently, the peptide of length 29 is encoded as a matrix or a vector of size 29 $\times$ 29.

BLOSUM62 encoding: BLOSUM62 (BLOck Substitution Matrix 62) [48], is a widely used substitution matrix in protein sequence alignment. The matrix assigns scores to pairs of amino acids based on their substitution frequency during evolution. Higher scores indicate more frequent substitutions, while lower scores indicate rare substitutions. In this study, we used the rows of the BLUSUM62 matrix to encode amino acids in the peptide. Consequently, the peptide of length 29 is encoded as a matrix or a vector of size 29 $\times$ 21.

### The structures of the plantNh-Kcr model

**Fig. 2:**
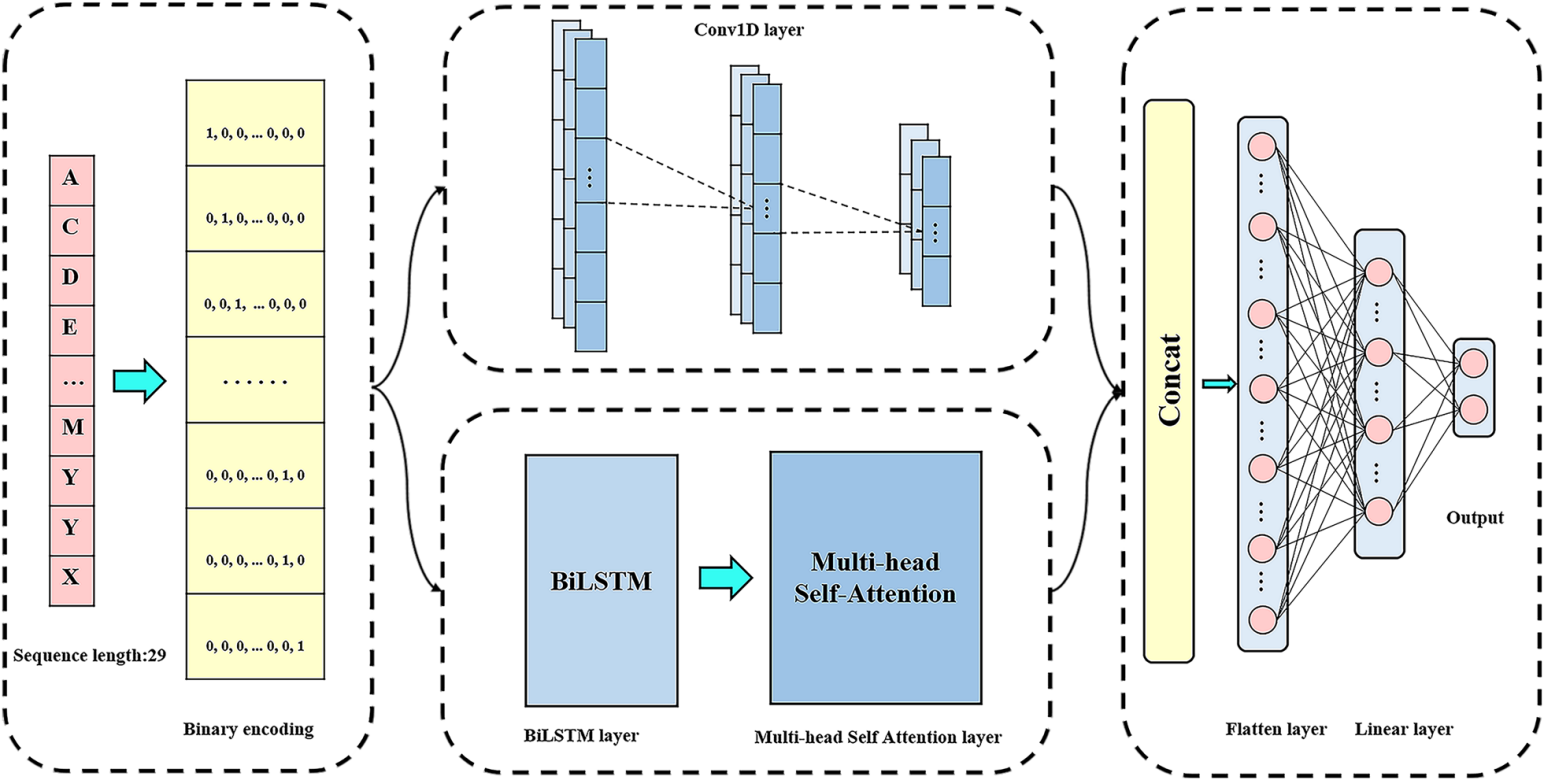
The architecture of the PlantNh-Kcr model

The structure of PlantNh-Kcr was determined as Fig. 2 after evaluating various encoding methods and model architectures. The model accepts a 29 × 21 matrix derived from binary encoding as input. This matrix feeds into two distinct layers. The first is a convolutional layer that is followed by two additional convolutional layers. The second layer is a BiLSTM layer that is succeeded by a MHSA layer. The outputs of the third convolutional layer and the MHSA layer are merged and flattened into a vector. The flatten layer is followed by a linear layer and an output layer. All the layers are described in detail below.

Input layer: The layer receives a 29 $\times$ 21 matrix as input.

Convolutional layers: The first convolutional layer has 21 input channels and 32 output channels, with a kernel size of 5 and a stride of 1. The second convolutional layer has 32 input channels and 32 output channels, and the third one has 32 input channels and 29 output channels. Both the latter two layers have a kernel size of 5 and a stride of 2. The outputs of each layer are activated using the ReLU function [49]. During training, to prevent overfitting, 30% of the output data from the three convolution layers are dropped respectively.

BiLSTM layer and MHSA layer: The input size of the BiLSTM layer is 21, and the output size is 128. The MHSA layer has an input size of 128 and eight attention heads. To prevent overfitting, dropout operations with ratios of 0.9 and 0.5 are applied after the BiLSTM layer and MHSA layer, respectively.

Flatten layer: The flatten layer flattens the concatenated outputs of the third convolutional layer and MHSA layer, resulting in a 3944-dimensional vector.

Linear layer: The input size of the linear layer is 3944 and the output size is 128. The output is activated using the ReLU function.

Output layer: The output layer has an input size of 128 and output size of 2. The two-dimensional output vector represents the probabilities of a sample being positive and negative, respectively.

### Focal loss

In this study, the training dataset has significantly more negative samples than positive samples, which would lead to a bias towards the negative samples during model training. To address this issue, we employed focal loss [50] as the loss function for the model. Focal loss reshapes the traditional cross-entropy loss function by introducing a modulating factor ${(1-{p}_{t})}^{\gamma }$. The mathematical formulas for focal loss are as follows:

1 $${p}_{t}=\left\{\begin{array}{l}p \qquad \quad if\, y=1\\ 1-p \quad \,otherwise.\end{array}\right. 0\le p\le 1$$

2 $${\alpha }_{t}=\left\{\begin{array}{l}\alpha \qquad \quad if\, y=1\\ 1-\alpha \quad \,otherwise.\end{array}\right. 0\le \alpha \le 1$$

3 $${\text{FL}}\left({p}_{t}\right)=-{\alpha }_{t}{\left(1-{p}_{t}\right)}^{\gamma }log({p}_{t}) \quad 0\le {\alpha }_{t}\le 1,\gamma \ge 0$$

where $p$ represents the probability for the sample to be class 1 (1 represents positive samples, while 0 represents negative samples). ${\alpha }_{t}$ represents the balanced weight factor for the sample. The modulating factor ${(1-{p}_{t})}^{\gamma }$ is used to adjust the weight of easy samples and hard samples, and $\upgamma$ indicates a tunable focusing parameter.

### Optimization of the model

The PlantNh-Kcr model was constructed and trained in a Python 3.9 and Pytorch 1.13.1 environment. Focal loss [50], with α set to 0.7 and γ to 1, was employed as the loss function. The model optimization was achieved using the Adam algorithm [51] with a learning rate of 0.001. The batch size of the input data during training was set to 256, and the number of training epochs was set to 50. To determine the optimal hyperparameters for the model, grid search was employed.

### Model evaluation

In bioinformatics, the evaluation of classification models often involves cross-validation and independent test to assess their generalization capabilities. Similar to previous studies [33, 35, 37, 38] that predicted Kcr sites, we employed five-fold cross-validation and independent tests to evaluate PlantNh-Kcr and other models. For this purpose, we prepared the training dataset and the test dataset. For five-fold cross-validation, the training dataset was evenly divided into five folds. Four folds were used to learn the model, while the remaining one was used to validate its performance. This process was repeated five times, ensuring that each fold was used once for validation. For independent test, the training dataset was used to build the model, and the test dataset was used to access its performance.

In bioinformatics, commonly used evaluation metrics for binary classification models include sensitivity (Sn), specificity (Sp), accuracy (ACC), F1-score, Matthews correlation coefficient (MCC), and area under the receiver operating characteristic (ROC) curve (AUC) [35, 38, 52, 53]. The mathematical formulas for Sn, Sp, ACC, and MCC are as follows:

4 $${\text{Sn}}=\frac{{\text{TP}}}{{\text{TP}}+{\text{FN}}}$$

5 $${\text{Sp}}=\frac{{\text{TN}}}{{\text{TN}}+{\text{FP}}}$$

6 $${\text{ACC}}=\frac{{\text{TP}}+{\text{TN}}}{{\text{TP}}+{\text{TN}}+{\text{FP}}+{\text{FN}}}$$

7 $${\text{F}}1-{\text{score}}=\frac{2\times {\text{TP}}}{2\times {\text{TP}}+{\text{FP}}+{\text{FN}}}$$

8 $${\text{MCC}}=\frac{\left({\text{TP}}\times {\text{TN}}\right)-\left({\text{FP}}\times {\text{FN}}\right)}{\sqrt{\left({\text{TP}}+{\text{FP}}\right)\left({\text{TP}}+{\text{FN}}\right)\left({\text{TN}}+{\text{FP}}\right)\left({\text{TN}}+{\text{FN}}\right)}}$$

In the above equations, TP, FP, TN, and FN represent the numbers of true positives, false positives, true negatives, and false negatives, respectively. Sn indicates the ability of the model to identify positive samples, with higher values indicating more accurate predictions for positive samples. Sp reflects the ability of the model to identify negative samples, with higher values indicating more accurate predictions for negative samples. F1-score provides a comprehensive measure of the model's performance in identifying positive samples, through balancing the counts of true positives, false positives, and false negatives. A higher F1-score indicates better performance. MCC considers both Sn and Sp, and ranges from − 1 to 1. A higher MCC value indicates better performance of the model. The ROC curve offers a graphical representation of the relationship between the true positive rate (TPR) and false positive rate (FPR) at different thresholds. TPR corresponds to Sn, while FPR equals one minus Sp. AUC represents the probability of a model ranking positive samples above negative samples. AUC is regarded as the most important metric in the evaluation of many bioinformatics models. The closer the ROC curve approaches the upper left corner, the closer the AUC value approaches 1, indicating a better classification performance of the model. In this study, samples with a predicted probability of being positive greater than 0.5 are classified as positive samples. The evaluation metrics of Sn, Sp, ACC, F1-score, and MCC are computed based on the fixed threshold of 0.5. We primarily use the ROC curve and its corresponding AUC value to compare the performance of different models. The ROC curve effectively visualizes the trade-off between Sn and Sp across various thresholds. This allows to compare models by considering their respective sensitivities at the same specificities, thus providing a more comprehensive evaluation.

To ensure the robustness of our model's performance, we conducted rigorous tests. For five-fold cross-validation, we calculated the mean and standard deviation of the metric values obtained from each fold. For independent test, we conducted 10 independent tests with different random seeds, and calculated the mean and standard deviation of the evaluated metrics.

## Results

### Conservation analysis of non-histone Kcr sites in plants

**Fig. 3:**
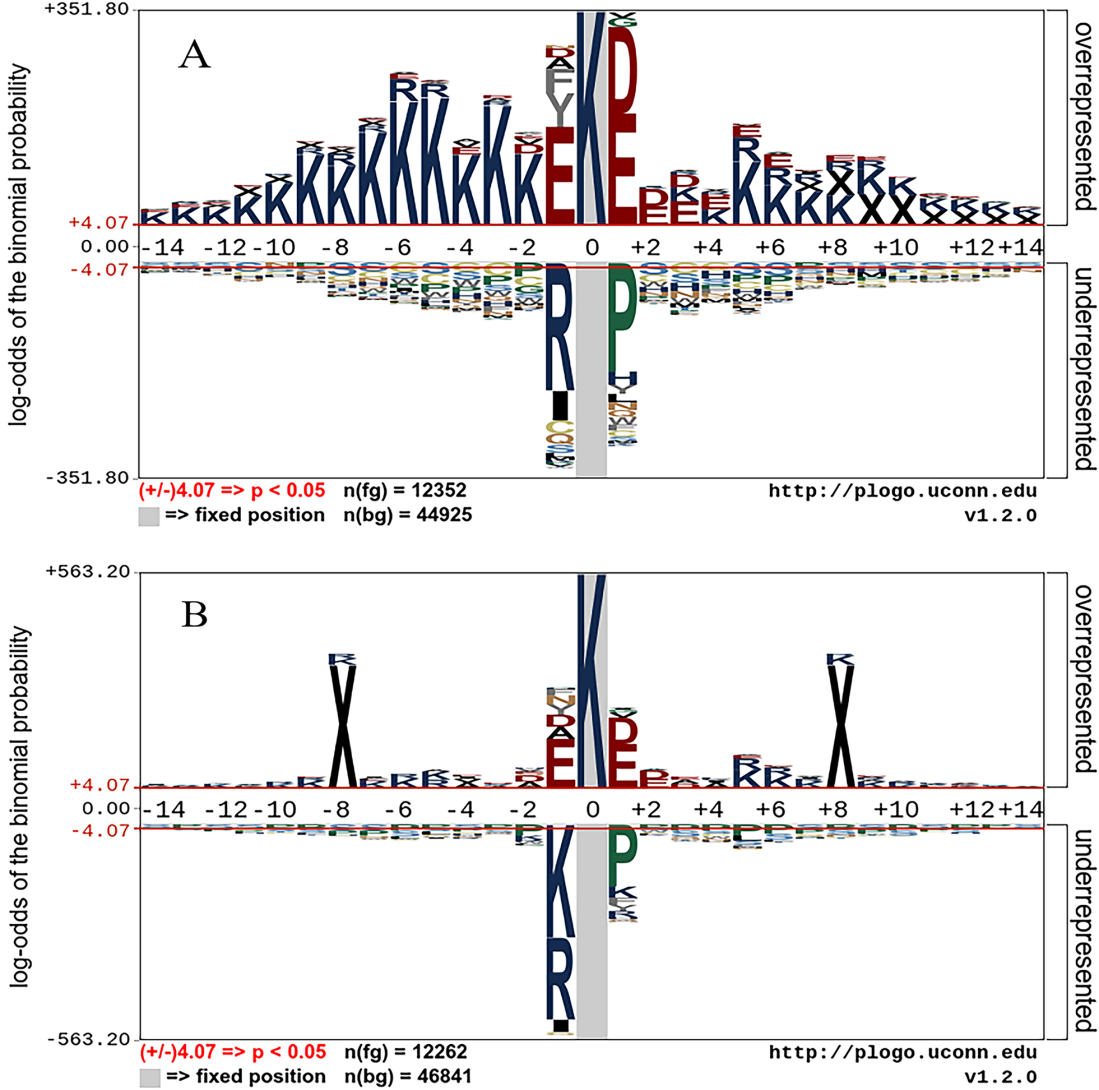
Sequence logo of Kcr sites on non-histone proteins. **A** Sequence logo for plants; **B** Sequence logo for humans

Kcr is a post-translational modification that plays a crucial role in various cellular processes. It has been observed that the evolution of Kcr sites exhibits conservation, which suggests that these sites have functional significance [35]. To further investigate the conservation of plant non-histone Kcr sites, we used the pLogo tool [54] to generate a sequence logo (Fig. 3A) using the merged training and test dataset. Significant disparities can be observed in the distribution of some amino acids such as K, D, E, R, and P, surrounding Kcr sites and non-Kcr sites. Notably, residues D and E are overrepresented at positions -1 and + 1. Residue K is prevalent at numerous positions, and it is more overrepresented on the left side of Kcr sites. Residues R and P are significantly underrepresented at positions − 1 and + 1, respectively. Previous research has identified specific motifs that are enriched around Kcr sites, including “EkxxxxxK”, “EkxxxK”, and “KxxxEK”, where x denotes any amino acid [35, 38]. These findings are consistent with our sequence logo, which indicates an overrepresentation of amino acid residues K and E in the vicinity of Kcr sites in plants. To further contextualize our findings, we also generated a sequence logo (Fig. 3B) for Kcr sites on non-histone proteins in humans. The resulting logo was based on the training dataset of nhKcr, a model developed for predicting Kcr sites on human non-histones [35]. Notably, the amino acid distribution observed around human Kcr sites exhibits similarity to that in plants; however, there were slight differences. For example, K is underrepresented at positions − 1 and + 1 for the human Kcr sites, which is not observed in plants. This observation highlights the need for developing a predictive model dedicated to plants.

### Performance of PlantNh-Kcr on five-fold cross-validation and independent tests

**Fig. 4:**
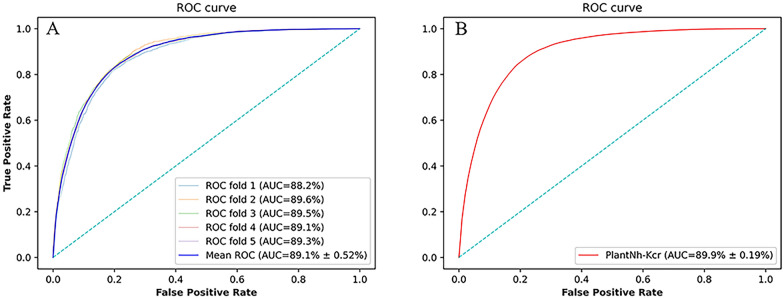
ROC curves of the PlantNh-Kcr model on five-fold cross-validation and independent tests. **A** The ROC curves on five-fold cross-validation; **B** The ROC curve on independent tests

To evaluate the performance of PlantNh-Kcr, we conducted a five-fold cross-validation and 10 independent tests. For cross-validation, the ROC curves for each fold were tightly clustered in the top left corner of the plot (Fig. 4A), indicating that the model has strong discriminatory power. The average AUC value are 0.891, which is significantly better than random prediction. The average values for Sn, Sp, ACC, F1-score and MCC are 0.821, 0.810, 0.812, 0.648 and 0.551 respectively (Table 2). For independent tests, PlantNh-Kcr also demonstrats strong performance, with the average AUC value of 0.899 (Fig. 4B) and the average values for Sn, Sp, ACC, F1-score, and MCC are 0.811, 0.833, 0.828, 0.665 and 0.572, respectively (Table 2).

**Table 2:** Metric values of the PlantNh-Kcr model on five-fold cross-validation and independent tests

Evaluation methods	Sn (%)	Sp (%)	ACC (%)	F1˗score (%)	MCC (%)	AUC (%)
Cross-validation	82.1 ± 2.36	81.0 ± 1.91	81.2 ± 1.04	64.8 ± 0.33	55.1 ± 0.54	89.1 ± 0.54
Independent test	81.1 ± 3.23	83.3 ± 2.09	82.8 ± 0.99	66.5 ± 0.50	57.2 ± 0.50	89.9 ± 0.19

**Fig. 5:**
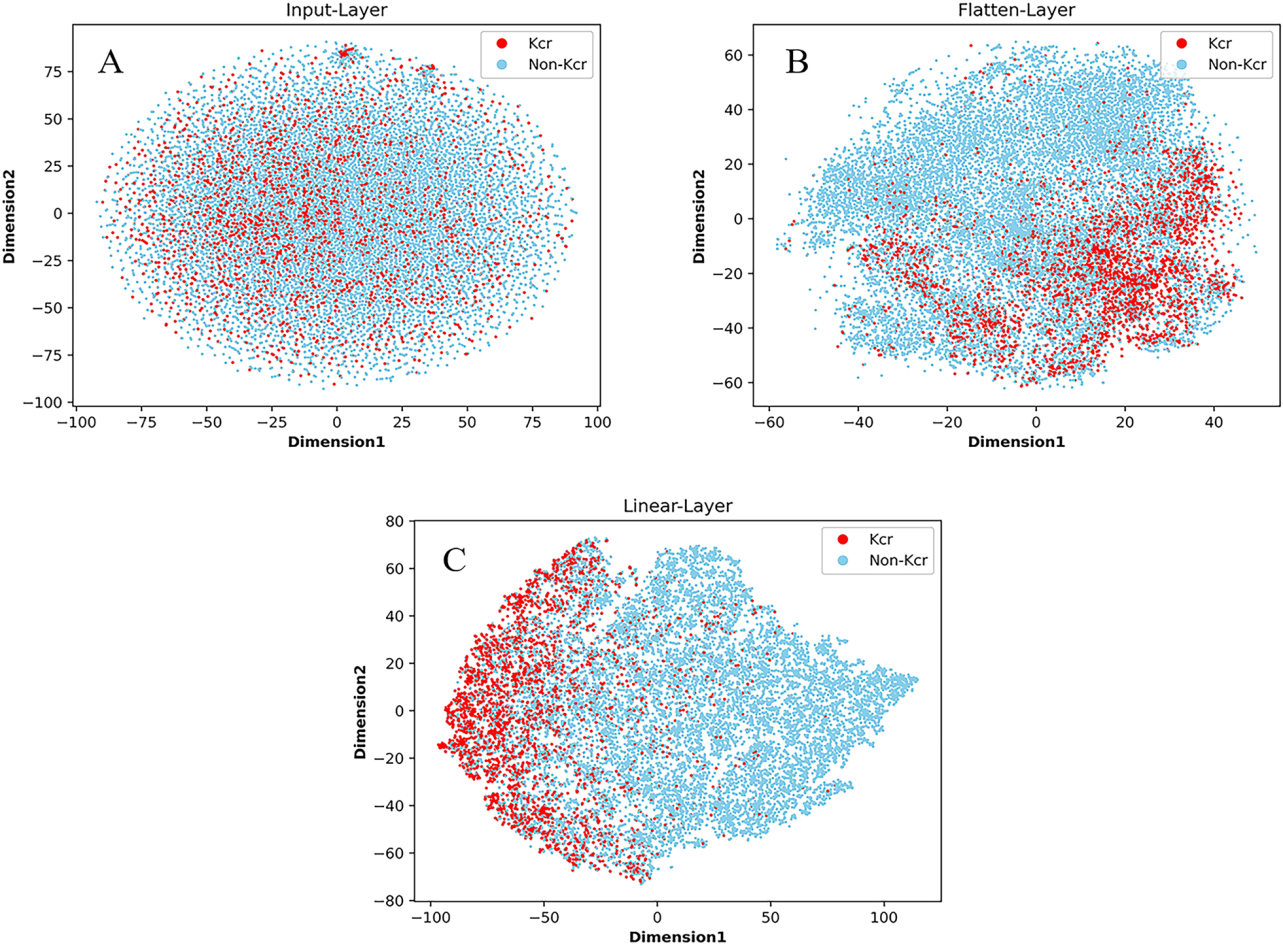
T-SNE visualization of test samples in PlantNh-Kcr layers. **A** The input layer; **B** The flatten layer; **C** The linear layer

To visualize the discriminatory power of PlantNh-Kcr, we utilized the training dataset to train a model and subsequently fed the samples in the test dataset to it. Then we used t-SNE [55] for dimensionality reduction and visualization of the input data in the input layer, the output data of the flatten layer, and the output data of the linear layer. The results are presented in Fig. 5. In this figure, the red and light blue dots represent Kcr and non-Kcr sites, respectively. It is evident from the input layer that Kcr and non-Kcr sites are intermingled. However, following the processing involving three convolutional layers, a BiLSTM layer, and a MHSA layer, most Kcr and non-Kcr sites are separated. This observation underscores the discriminatory capability of these layers in effectively distinguishing between Kcr and non-Kcr sites. Subsequently, after the linear layer, Kcr sites are predominantly clustered in the left region, forming a distinct boundary from non-Kcr sites. This outcome highlights the model’s prowess in accurately classifying Kcr sites.

### Comparison with conventional machine learning models and deep learning models

To further demonstrate the superior performance and robustness of PlantNh-Kcr, we conducted a comparative analysis with several well-established conventional machine learning models and deep learning models. The detailed information about these models is provided in Additional file 1.

**Fig. 6:**
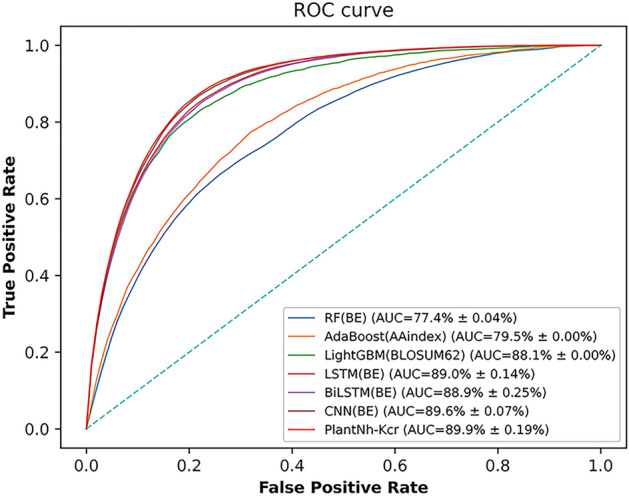
ROC curves of different models on independent tests

In this study, we utilized three conventional machine learning methods including RF [21], AdaBoost [56], and LightGBM [24]. Additionally, five commonly used encodings were employed, including BE, ACC, EGAAC, AAindex, and BLOUSUM62. Each encoding was fed into each conventional machine learning model for training. The specific results for each combination are summarized in Tables 3, 4. For both five-fold cross-validation and independent tests, RF, AdaBoost, and LightGBM get the largest AUC values, when BE, AAindex, and BLOSUM62 encodings were used as input features, respectively. Among the three models, the LightGBM model emerged as the leader with average AUC values of 0.869 and 0.881 on five-fold cross-validation and independent tests, respectively. However, it lagged behind PlantNh-Kcr in terms of performance. To visually compare the models, Fig. 6 presents the ROC curves on independent tests. Notably, The ROC curve of PlantNh-Kcr is above those of other models. This observation indicates that at the same FPR, PlantNh-Kcr exhibits the highest TPR, indicating its superior ability to correctly identify positive samples compared to other models when predicting the same number of false positives.

**Table 3:** Metric values of different models on five-fold cross-validation

Classifiers	Encodings	Sn (%)	Sp (%)	ACC (%)	F1˗score (%)	MCC (%)	AUC (%)
RF	^**a**^**BE**	**69.4 **$\pm$** 1.12**	**71.3 **$\pm$** 0.94**	**70.9** $\pm$ **0.76**	**50.1** $\pm$ **0.99**	**34.4 **$\pm$** 1.29**	**77.5 **$\pm$** 0.77**
	AAC	70.5 $\pm$ 1.89	64.8 $\pm$ 0.99	66.0 $\pm$ 0.55	44.6 $\pm$ 0.78	29.1 $\pm$ 1.02	74.5 $\pm$ 0.63
	EGAAC	70.9 $\pm$ 1.47	59.3 $\pm$ 1.44	61.8 $\pm$ 0.87	43.8 $\pm$ 0.82	24.7 $\pm$ 0.67	71.1 $\pm$ 0.30
	AAindex	74.1 $\pm$ 0.83	60.9 $\pm$ 1.41	63.7 $\pm$ 0.98	46.2 $\pm$ 0.58	28.6 $\pm$ 0.78	75.2 $\pm$ 0.56
	BLOSUM62	70.1 $\pm$ 1.04	66.8 $\pm$ 0.53	67.5 $\pm$ 0.30	47.6 $\pm$ 0.44	30.6 $\pm$ 0.60	75.5 $\pm$ 0.50
AdaBoost	BE	24.3 $\pm$ 1.28	95.4 $\pm$ 0.17	80.5 $\pm$ 0.51	34.4 $\pm$ 1.60	28.6 $\pm$ 1.82	79.0 $\pm$ 0.60
	AAC	17.6 $\pm$ 0.79	95.3 $\pm$ 0.28	79.0 $\pm$ 0.32	26.0 $\pm$ 0.76	20.1 $\pm$ 0.52	75.8 $\pm$ 0.63
	EGAAC	2.40 $\pm$ 0.30	99.4 $\pm$ 0.17	79.0 $\pm$ 0.28	4.70 $\pm$ 0.54	7.60 $\pm$ 1.05	71.5 $\pm$ 0.66
	**AAindex**	**25.4 **$\pm$** 0.86**	**95.3 **$\pm$** 0.36**	**80.6 **$\pm$** 0.28**	**35.6** $\pm$ **1.09**	**29.5 **$\pm$** 1.43**	**79.4 **$\pm$** 0.49**
	BLOSUM62	24.2 $\pm$ 0.79	95.6 $\pm$ 0.30	80.6 $\pm$ 0.26	34.4 $\pm$ 0.80	28.8 $\pm$ 0.77	78.9 $\pm$ 0.55
LightGBM	BE	68.6 $\pm$ 1.30	85.0 $\pm$ 0.23	81.5 $\pm$ 0.47	60.9 $\pm$ 1.00	49.5 $\pm$ 1.32	85.6 $\pm$ 0.52
	AAC	67.3 $\pm$ 1.03	73.5 $\pm$ 0.74	72.2 $\pm$ 0.51	50.4 $\pm$ 0.66	34.8 $\pm$ 0.77	78.0 $\pm$ 0.81
	EGAAC	70.3 $\pm$ 0.48	59.4 $\pm$ 0.85	61.7 $\pm$ 0.67	43.6 $\pm$ 0.39	24.3 $\pm$ 0.75	70.5 $\pm$ 0.62
	AAindex	65.1 $\pm$ 1.13	88.0 $\pm$ 0.31	83.2 $\pm$ 0.32	61.9 $\pm$ 0.81	51.3 $\pm$ 0.99	86.6 $\pm$ 0.23
	**BLOSUM62**	**66.8 **$\pm$** 0.64**	**87.2 **$\pm$** 0.60**	**83.0 **$\pm$** 0.50**	**62.3** $\pm$ **0.93**	**51.7 **$\pm$** 1.17**	**86.9 **$\pm$** 0.54**
LSTM	**BE**	**78.3 **$\pm$** 2.23**	**81.7 **$\pm$** 2.08**	**81.0 **$\pm$** 1.20**	**63.4** $\pm$ **0.86**	**53.0 **$\pm$** 0.99**	**88.2 **$\pm$** 0.12**
	WE	74.8 $\pm$ 0.79	81.8 $\pm$ 0.47	80.4 $\pm$ 0.24	61.6 $\pm$ 0.37	50.3 $\pm$ 0.36	86.5 $\pm$ 0.37
	AAindex	76.6 $\pm$ 4.05	83.1 $\pm$ 2.18	81.8 $\pm$ 0.97	63.8 $\pm$ 0.94	53.5 $\pm$ 1.15	88.0 $\pm$ 0.59
	BLOSUM62	72.3 $\pm$ 3.92	84.7 $\pm$ 2.36	82.1 $\pm$ 1.18	63.0 $\pm$ 1.26	52.3 $\pm$ 1.50	87.6 $\pm$ 0.43
BiLSTM	**BE**	**76.9 **$\pm$** 3.03**	**82.1 **$\pm$** 2.71**	**81.0 **$\pm$** 1.50**	**63.1** $\pm$ **1.13**	**52.6 **$\pm$** 1.22**	**88.0 **$\pm$** 0.25**
	WE	74.4 $\pm$ 2.67	81.9 $\pm$ 130	80.3 $\pm$ 0.50	61.4 $\pm$ 0.55	50.1 $\pm$ 0.62	86.6 $\pm$ 0.54
	AAindex	77.6 $\pm$ 2.28	81.7 $\pm$ 1.25	80.9 $\pm$ 0.56	63.0 $\pm$ 0.55	52.4 $\pm$ 0.65	88.1 $\pm$ 0.30
	BLOSUM62	81.1 $\pm$ 3.10	78.4 $\pm$ 2.95	79.0 $\pm$ 1.70	62.0 $\pm$ 0.86	51.3 $\pm$ 1.01	87.7 $\pm$ 0.31
CNN	**BE**	**80.9 **$\pm$** 1.78**	**81.5 **$\pm$** 0.92**	**81.0 **$\pm$** 0.50**	**64.2** $\pm$ **0.89**	**54.1 **$\pm$** 1.04**	**88.8 **$\pm$** 0.34**
	WE	80.3 $\pm$ 2.59	81.6 $\pm$ 1.95	81.4 $\pm$ 1.13	64.4 $\pm$ 1.25	54.4 $\pm$ 1.43	88.6 $\pm$ 0.47
	AAindex	78.3 $\pm$ 4.65	82.6 $\pm$ 2.94	81.7 $\pm$ 1.40	64.3 $\pm$ 0.87	54.2 $\pm$ 0.90	88.5 $\pm$ 0.64
	BLOSUM62	77.3 $\pm$ 4.29	83.0 $\pm$ 2.75	81.9 $\pm$ 1.31	64.2 $\pm$ 0.82	54.0 $\pm$ 0.96	88.6 $\pm$ 0.32
PlantNh-Kcr	**BE**	**82.1 **$\pm$** 2.36**	**81.0** $\pm$ **1.91**	**81.2** $\pm$ **1.04**	**64.8 **$\pm$** 0.33**	**55.1** $\pm$ **0.54**	**89.1** $\pm$ **0.54**

^a^Bold indicates the best performance for the classifier

**Table 4:** Metric values of different models on independent test

Classifiers	Encodings	Sn (%)	Sp (%)	ACC (%)	F1˗score (%)	MCC (%)	AUC (%)
RF	**BE**	**69.6 **$\pm$** 0.30**	**70.7 **$\pm$** 0.41**	**70.5** $\pm$ **0.27**	**49.8** $\pm$ **0.15**	**33.9 **$\pm$** 0.22**	**77.4 **$\pm$** 0.04**
	AAC	70.5 $\pm$ 0.32	65.1 $\pm$ 0.20	66.2 $\pm$ 0.15	46.8 $\pm$ 0.18	29.4 $\pm$ 0.28	74.6 $\pm$ 0.06
	EGAAC	70.5 $\pm$ 0.71	60.9 $\pm$ 0.54	62.9 $\pm$ 0.29	44.4 $\pm$ 0.10	25.6 $\pm$ 0.17	71.1 $\pm$ 0.01
	AAindex	74.5 $\pm$ 0.40	60.0 $\pm$ 043	63.0 $\pm$ 0.27	45.9 $\pm$ 0.11	28.2 $\pm$ 0.17	75.2 $\pm$ 0.10
	BLOSUM62	70.0 $\pm$ 0.42	66.4 $\pm$ 0.40	67.1 $\pm$ 0.27	47.2 $\pm$ 0.20	30.1 $\pm$ 0.30	75.4 $\pm$ 0.11
AdaBoost	BE	23.5 $\pm$ 0.00	95.4 $\pm$ 0.00	80.3 $\pm$ 0.00	33.8 $\pm$ 0.00	27.8 $\pm$ 0.00	78.9 $\pm$ 0.00
	AAC	18.0 $\pm$ 0.00	95.6 $\pm$ 0.00	79.3 $\pm$ 0.00	26.8 $\pm$ 0.00	23.1 $\pm$ 0.00	76.2 $\pm$ 0.00
	EGAAC	2.60 $\pm$ 0.00	99.4 $\pm$ 0.00	79.0 $\pm$ 0.00	5.00 $\pm$ 0.00	7.90 $\pm$ 0.00	71.5 $\pm$ 0.00
	**AAindex**	**25.8 **$\pm$** 0.00**	**95.2 **$\pm$** 0.00**	**80.6** $\pm$ **0.00**	**35.0** $\pm$ **0.00**	**29.5 **$\pm$** 0.00**	**79.5 **$\pm$** 0.00**
	BLOSUM62	23.1 $\pm$ 0.00	95.3 $\pm$ 0.00	80.1 $\pm$ 0.00	32.9 $\pm$ 0.00	26.9 $\pm$ 0.00	79.0 $\pm$ 0.00
LightGBM	BE	71.9 $\pm$ 0.00	84.0 $\pm$ 0.00	81.4 $\pm$ 0.00	62.0 $\pm$ 0.00	50.8 $\pm$ 0.00	86.6 $\pm$ 0.00
	AAC	69.0 $\pm$ 0.00	72.2 $\pm$ 0.00	71.5 $\pm$ 0.00	50.5 $\pm$ 0.00	34.9 $\pm$ 0.00	78.4 $\pm$ 0.00
	EGAAC	72.4 $\pm$ 0.00	59.0 $\pm$ 0.00	61.9 $\pm$ 0.00	44.4 $\pm$ 0.00	25.7 $\pm$ 0.00	71.1 $\pm$ 0.00
	AAindex	69.4 $\pm$ 0.00	86.9 $\pm$ 0.00	83.2 $\pm$ 0.00	63.5 $\pm$ 0.00	53.0 $\pm$ 0.00	87.6 $\pm$ 0.00
	**BLOSUM62**	**71.7 **$\pm$** 0.00**	**86.2 **$\pm$** 0.00**	**83.2 **$\pm$** 0.00**	**64.2** $\pm$ **0.00**	**53.8 **$\pm$** 0.00**	**88.1 **$\pm$** 0.00**
LSTM	**BE**	**79.7 **$\pm$** 5.22**	**82.2 **$\pm$** 3.29**	**81.7 **$\pm$** 1.54**	**64.7** $\pm$ **0.66**	**54.9 **$\pm$** 0.60**	**89.0 **$\pm$** 0.14**
	WE	75.2 $\pm$ 1.82	83.4 $\pm$ 1.32	81.7 $\pm$ 0.62	63.3 $\pm$ 0.40	52.7 $\pm$ 0.51	87.5 $\pm$ 0.21
	AAindex	79.0 $\pm$ 4.48	82.5 $\pm$ 3.21	81.7 $\pm$ 1.61	64.6 $\pm$ 0.77	54.6 $\pm$ 0.72	88.7 $\pm$ 0.32
	BLOSUM62	75.5 $\pm$ 4.72	83.8 $\pm$ 2.88	82.0 $\pm$ 1.33	63.9 $\pm$ 0.68	53.6 $\pm$ 0.82	88.5 $\pm$ 0.43
BiLSTM	**BE**	**75.8 **$\pm$** 3.49**	**84.3 **$\pm$** 1.77**	**82.5 **$\pm$** 0.71**	**64.6** $\pm$ **0.50**	**54.5 **$\pm$** 0.67**	**88.9 **$\pm$** 0.25**
	WE	77.5 $\pm$ 2.79	81.0 $\pm$ 0.20	80.3 $\pm$ 1.03	62.3 $\pm$ 0.60	51.5 $\pm$ 0.73	87.4 $\pm$ 0.28
	AAindex	79.3 $\pm$ 3.45	82.2 $\pm$ 2.59	81.6 $\pm$ 1.37	64.5 $\pm$ 0.88	54.5 $\pm$ 0.96	88.9 $\pm$ 0.26
	BLOSUM62	75.5 $\pm$ 7.16	83.5 $\pm$ 4.16	81.8 $\pm$ 1.81	63.7 $\pm$ 0.58	53.5 $\pm$ 0.50	88.7 $\pm$ 0.13
CNN	**BE**	**82.1 **$\pm$** 1.08**	**82.2 **$\pm$** 0.80**	**82.1 **$\pm$** 0.43**	**66.0** $\pm$ **0.33**	**56.5 **$\pm$** 0.38**	**89.6 **$\pm$** 0.07**
	WE	79.0 $\pm$ 2.13	83.4 $\pm$ 1.33	82.4 $\pm$ 0.54	65.4 $\pm$ 0.48	55.6 $\pm$ 0.63	89.1 $\pm$ 0.16
	AAindex	82.1 $\pm$ 3.27	81.2 $\pm$ 2.06	81.4 $\pm$ 0.96	65.0 $\pm$ 0.39	55.3 $\pm$ 0.38	89.1 $\pm$ 0.14
	BLOSUM62	79.2 $\pm$ 4.92	83.1 $\pm$ 2.88	82.3 $\pm$ 1.26	65.3 $\pm$ 0.43	55.6 $\pm$ 0.38	89.4 $\pm$ 0.11
PlantNh-Kcr	**BE**	**81.1** $\pm$ **3.23**	**83.3** $\pm$ **2.09**	**82.8** $\pm$ **0.99**	**66.5** $\pm$ **0.50**	**57.2** $\pm$ **0.50**	**89.9** $\pm$ **0.19**

^a^Bold indicates the best performance for the classifier

Three networks including LSTM network, BiLSTM network, and CNN were used to compare with PlantNh-Kcr. The inputs for these networks encompassed BE, WE, AAindex, and BLOSUM62 encodings. The specific metric values for each network are detailed in Tables 3,  4. Interestingly, all three networks perform best when using BE as input. On five-fold cross-validation, the maximum average AUC values achieved by the LSTM, BiLSTM, and CNN networks are 0.882, 0.880 and 0.888, respectively. Similarly, on independent tests, the maximum average AUC values of these networks are 0.890, 0.889, and 0.896, respectively. However, the performance of the three networks is still inferior to PlantNh-Kcr.

### Comparison with existing models for Kcr site prediction on non-histones

To further demonstrate the performance of our model, we conducted a comparative analysis with four other models: nhKcr, iKcr_CNN, CapsNh-Kcr, and DeepKcrot, all designed to predict Kcr sites on non-histones. nhKcr, iKcr_CNN and CapsNh-Kcr predict Kcr sites in human. The nhKcr model integrated BE, AAindex encoding and BLOSUM62 encoding as input features and employed a CNN architecture. The iKcr_CNN model employed a CNN architecture and utilized a focal loss function for optimization. CapsNh-Kcr employed a CNN-based capsule network strategy. DeepKcrot predicted Kcr sites in four species including human, rice, papaya and tabacum. It utilized CNN with WE encoding as input features.

**Fig. 7:**
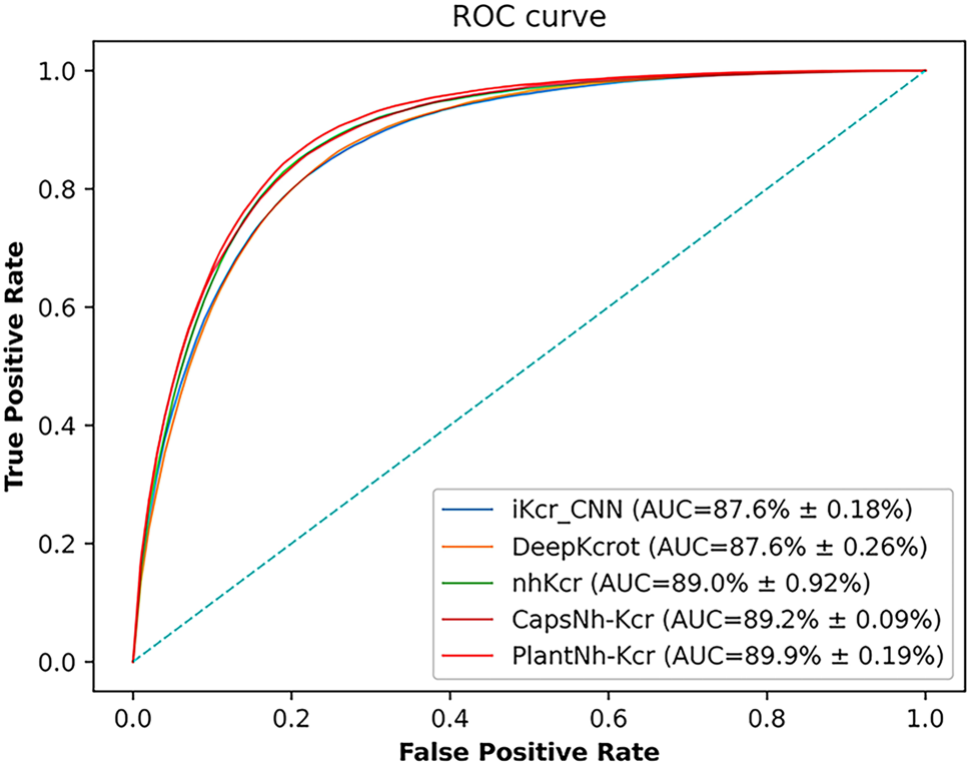
ROC curves of PlantNh-Kcr and the other four models

For nhKcr, iKcr_CNN and CapsNh-Kcr, we downloaded their source codes. For DeepKcrot, we rewrote its code due to the unavailability of its web server. We applied focal loss to nhKcr and DeepKcrot because they didn’t address the data imbalance issue in their original source codes. We then trained the four models using the training dataset and evaluated their performance on the test set. The prediction performance of the four models is shown in Fig. 7 and Table 5. The average AUC values are 0.876, 0.876, 0.890, and 0.892, respectively, which are lower than that of PlantNh-Kcr. This again underscores the superior performance of PlantNh-Kcr.

**Table 5:** Metric values of PlantNh-Kcr and the other four models

Models	Sn (%)	Sp (%)	ACC (%)	F1˗score (%)	MCC (%)	AUC (%)
iKcr_CNN	77.2 $\pm$ 1.07	82.0 $\pm$ 0.79	81.0 $\pm$ 0.45	63.1 $\pm$ 0.39	52.5 $\pm$ 0.51	87.6 $\pm$ 0.18
DeepKcrot	82.8 $\pm$ 1.53	77.9 $\pm$ 1.46	78.9 $\pm$ 0.85	62.3 $\pm$ 0.56	51.9 $\pm$ 0.59	87.6 $\pm$ 0.26
nhKcr	87.6 $\pm$ 2.42	77.3 $\pm$ 3.26	79.4 $\pm$ 3.26	64.3 $\pm$ 1.61	55.1 $\pm$ 1.63	89.0 $\pm$ 0.92
CapsNh-Kcr	76.4 $\pm$ 3.59	84.3 $\pm$ 1.59	83.1 $\pm$ 0.71	65.5 $\pm$ 0.47	55.6 $\pm$ 0.58	89.2 $\pm$ 0.09
PlantNh-Kcr	81.1 $\pm$ 3.23	83.3 $\pm$ 2.09	82.8 $\pm$ 0.99	66.5 $\pm$ 0.50	57.2 $\pm$ 0.50	89.9 $\pm$ 0.19

### Ablation study

To assess the effect of each component in the PlantNh-Kcr model on prediction performance, we conducted an ablation study. In this study, we removed the linear layer, CNN, MHSA, and BiLSTM + MHSA individually from the model and evaluated the prediction performance on independent tests. The results are summarized in Table 6. Removing the linear layer and CNN individually resulted in a decrease of 1.1% and 1.2% in AUC values, respectively. This suggests that these two components have a certain impact on the overall performance of the model. On the other hand, removing MHSA and BiLSTM + MHSA individually resulted in a decrease of 0.5% and 0.3% in AUC values, respectively, indicating that these components have a smaller impact on performance compared to the linear layer and CNN. Overall, our results demonstrate that each component in the PlantNh-Kcr model contributes to its prediction performance. Removing any module from the model will result in a decrease in performance, indicating that each module is essential for achieving optimal performance.

**Table 6:** Prediction performance of models in the ablation study

Model	Sn (%)	Sp (%)	ACC (%)	F1˗score (%)	MCC (%)	AUC (%)
Removing linear layer	90.0 $\pm$ 1.19	70.7 $\pm$ 2.24	74.8 $\pm$ 1.56	60.1 $\pm$ 1.27	50.2 $\pm$ 1.46	88.8 $\pm$ 0.32
Removing CNN	78.0 $\pm$ 4.48	82.6 $\pm$ 3.00	81.6 $\pm$ 1.50	64.1 $\pm$ 0.92	53.9 $\pm$ 1.09	88.7 $\pm$ 0.44
Removing MHSA	79.2 $\pm$ 2.33	84.5 $\pm$ 1.45	83.4 $\pm$ 0.70	66.7 $\pm$ 0.51	57.3 $\pm$ 0.64	89.4 $\pm$ 0.44
Removing BiLSTM + MHSA	82.1 $\pm$ 1.08	82.2 $\pm$ 0.80	82.1 $\pm$ 0.43	66.0 $\pm$ 0.33	56.5 $\pm$ 0.38	89.6 $\pm$ 0.07
PlantNh-Kcr	81.1 $\pm$ 3.23	83.3 $\pm$ 2.09	82.8 $\pm$ 0.99	66.5 $\pm$ 0.50	57.2 $\pm$ 0.50	89.9 $\pm$ 0.19

### The performance of PlantNh-Kcr on independent tests for each plant

**Fig. 8:**
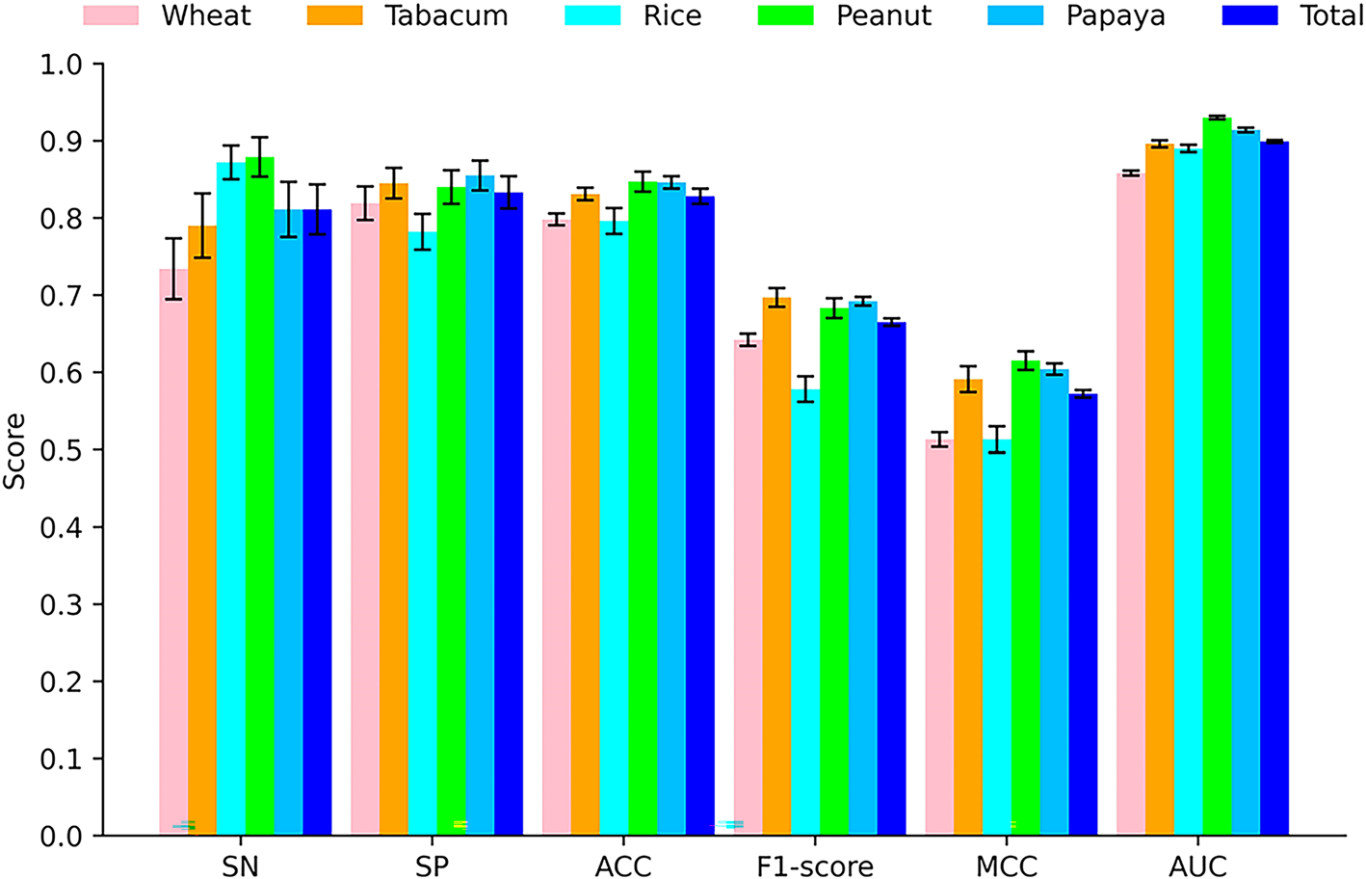
Metric values on independent tests for different plants

In this study, we collected non-histone Kcr sites from different types of plants. Given the potential species-specific impact on these sites, it’s necessary to assess the generalizability of our predictive model across diverse plant species. Therefore, we studied the performance of our model for each species on independent tests. Table 7 details the evaluation metrics for each species, which are further visualized in Fig. 8 as a bar chart.

**Table 7:** Performance of PlantNh-Kcr for different plants

Species	Sn (%)	Sp (%)	ACC (%)	F1-score (%)	MCC (%)	AUC (%)
Wheat	73.4 ± 3.94	81.9 ± 2.19	79.8 ± 0.76	64.2 ± 0.80	51.3 ± 0.93	85.8 ± 0.32
Tabacum	79.0 ± 4.16	84.5 ± 1.98	83.1 ± 0.81	69.7 ± 1.22	59.1 ± 1.67	89.6 ± 0.46
Rice	87.2 ± 2.18	78.2 ± 2.32	79.6 ± 1.68	57.8 ± 1.65	51.3 ± 1.69	89.0 ± 0.49
Peanut	87.9 ± 2.54	84.0 ± 2.17	84.7 ± 1.31	68.3 ± 1.29	61.5 ± 1.21	93.0 ± 0.21
Papaya	81.1 ± 3.58	85.5 ± 1.94	84.6 ± 0.81	69.2 ± 0.57	60.4 ± 0.75	91.4 ± 0.30

The results indicate that the prediction performance of the model varies slightly across different species. Notably, peanuts and papaya exhibit particularly strong performance, with average AUC values of 0.930 and 0.914, respectively. The model also demonstrates good performance for tabacum and rice, with average AUC values of 0.896 and 0.890, respectively. However, wheat exhibits slightly lower performance compared to other species, with an average AUC value of 0.858. This may be attributed to species-specific characteristics.

To study the performance of species-specific models, we developed individual models for each plant using samples from the corresponding species in the training dataset. We then evaluated these models using samples from the corresponding species in the test set. The performance of these models on five metrics are shown in Table 8. Notably, the peanut-specific and papaya-specific models exhibit the best performance, with average AUC values of 0.920 and 0.902, respectively. In contrast, the species-specific models for rice, tabacum, and wheat exhibit relatively poorer performance. This can be attributed to the smaller training set size for rice and tabacum and potential species-specific characteristics affecting crotonylation patterns in wheat. When compared with the general model’s performance in Table 7, the species-specific models underperform. This finding underscores the advantage of integrating data from diverse species to train a general predictive model for plant non-histone Kcr sites.

**Table 8:** Performance of species-specific models on independent tests

Species	Sn (%)	Sp (%)	ACC (%)	F1-score (%)	MCC (%)	AUC (%)
Wheat	73.3 ± 5.12	77.6 ± 4.26	76.5 ± 1.95	60.7 ± 0.53	46.2 ± 0.86	83.1 ± 0.36
Tabacum	72.4 ± 2.28	77.1 ± 1.06	76.0 ± 0.43	59.7 ± 0.77	44.7 ± 1.06	82.7 ± 0.77
Rice	66.2 ± 5.53	83.2 ± 3.46	80.4 ± 2.07	52.8 ± 1.11	43.2 ± 1.31	83.6 ± 0.80
Peanut	83.7 ± 2.55	84.8 ± 1.58	84.6 ± 0.84	67.1 ± 0.65	59.6 ± 0.62	92.0 ± 0.17
Papaya	84.4 ± 2.90	80.5 ± 2.26	81.4 ± 1.20	66.0 ± 0.85	56.6 ± 0.93	90.2 ± 0.19

## Discussion

The PlantNh-Kcr exhibits superior performance. However, there are still some issues that need to be considered.

First, our model PlantNh-Kcr contains three convolutional layers, which can effectively capture local patterns in protein sequences. Careful consideration must be given to the kernel size and the step size, as well as the number of convolution kernels. Too few or too many convolution kernels can lead to information loss or overfitting, respectively, which can impact model performance. Furthermore, when utilizing the convolutional layer to process long protein sequences, there is a risk of losing global contextual information. This can be a limiting factor in the predictive accuracy of the model. To address this issue, stacking multiple convolutional layers and effectively integrating their outputs can compensate for the loss of global context. By doing so, the model can achieve a more comprehensive understanding of the protein sequences, ultimately leading to improved predictive performance.

Second, multiple encodings were described in the paper, such as BE, WE encoding, AAindex encoding, and BLOSUM62 encoding. The PlantNh-Kcr model only utilize BE as input features. We have attempted to integrate multiple encodings as input features of the model, but failed to improve the performance. This may be because these features have poor complementarity.

Third, there are far more negative samples than positive samples in our training set. This imbalance can significantly influence model training, biasing it towards the negative samples. To address this issue, three methods were employed: up-sampling the positive samples, down-sampling the negative samples, and utilizing the focal loss function. Among these methods, the focal loss function presented the best prediction performance, and improved the ability of the model to correctly predict positive samples. We believe that dataset imbalance remains a potential problem that needs to be addressed in bioinformatics.

## Conclusion

In this study, we compiled a large dataset of non-histone Kcr sites from five different plant species. Using this dataset, we developed a deep learning model called PlantNh-Kcr to predict non-histone Kcr sites in plants. The model’s architecture integrates CNN, LSTM, and attention mechanism, utilizing BE as its primary input features. Notably, the model exhibits satisfactory performance on both five-fold cross-validation and independent tests, outperforming several other models. In addition, there are minor variations in prediction performance across different plant species, a general predictive model demonstrates superior performance compared to species-specific models. We believe that the PlantNh-Kcr model offers a valuable contribution to addressing challenges and advancing the study of plant Kcr sites. We also believe that as more Kcr sites are experimentally determined and as deep learning techniques continue to develop, we will see the emergence of more high-performance models for predicting Kcr sites.

### Supplementary Information

**Additional file 1.** Detailed information about the conventional machine learning models.

## Data Availability

The source code and datasets are publicly available at https://github.com/jiangyanming-individual/PlantNh-Kcr**.**
