# Supplementary material for: PlantNh-Kcr: a deep learning model for predicting non-histone crotonylation sites in plants
Source: Plant Methods. 2024 Feb 15;20:28. doi: 10.1186/s13007-024-01157-8 (PMC10870457; doi:10.1186/s13007-024-01157-8)
Supplement: Supplementary file 1 — Additional file 1. Detailed information about the conventional machine learning models. [file 13007_2024_1157_MOESM1_ESM.docx]

**PlantNh-Kcr: A deep learning model for predicting non-histone crotonylation sites in plants**

**Yanming Jiang^a^, Renxiang Yan^b, c^, Xiaofeng Wang****^a, *^**

*^a^College of Mathematics and Computer Sciences, Shanxi Normal University, Taiyuan 030031, China*

*^b^The Key Laboratory of Marine Enzyme Engineering of Fujian Province, Fuzhou University, Fuzhou* *350002, China*

*^c^College of Biological Science and Engineering, Fuzhou University, Fuzhou, 350002, China*

*Corresponding author: Xiaofeng Wang; Email: [wangxf@sxnu.edu.cn](mailto:wangxf@sxnu.edu.cn)

**Additional Information**

**Conventional machine learning algorithms**

***Random forest (RF)***

We implemented the random forest [1] algorithm using the scikit-learn package [2] in Python. The hyperparameters were set as follows: maximum number of n_estimators: 500, maximum depth: 10, and maximum leaf nodes: 10. To address the problem of data imbalance, the class_weight parameter was set to ‘balanced’.

***Adaptive Boosting (AdaBoost)***

We implemented the AdaBoost [3] algorithm also using the scikit-learn package in Python. The hyperparameters were set as follows: maximum number of n_estimators: 100.

***Light Gradient Boosted Machine (LightGBM)***

We employed the LightGBM package to implement the Light Gradient Boosted Machine algorithm [4-6] in Python. The hyperparameters were tuned as follows: maximum number of n_estimators: 500, maximum depth: 15, and learning_rate: 0.1. To tackle data imbalance, the class_weight parameter was set to ‘balanced’.

**Deep learning algorithms**

***Long short-term memory (LSTM)***

We implemented the LSTM network [7] using the PyTorch [8] package. Specifically, the input layer was followed by two LSTM layers. The sizes of the two layers were set to 64. The second LSTM layer was followed by a linear layer with 64 neurons and an output layer with two neurons. To prevent overfitting, a dropout operation with a rate of 0.5 was implemented for the output of the LSTM layers. During the training process, focal loss [9] was used as the loss function. The batch size for input data was set to 256. The number of the training epochs was set to 50. The learning rate was set to 0.001.

***Bidirectional long short-term memory (BiLSTM)***

We implemented the BiLSTM network [10] using the Pytorch framework. The network connected the input layer with two BiLSTM layers, and the hidden size was set to 64. After the BiLSTM layer, we connected a linear layer with 128 neurons and an output layer with two neurons. To prevent overfitting, dropout operation with rate of 0.5 and 0.9 was implemented for the output of the BiLSTM layer. During the training process, focal loss was used as the loss function. The batch size for input data was set to 256. The number of the training epochs was set to 50. The learning rate was set to 0.001. Adam [11] optimizer was used to tune the model parameters.

***Convolutional neural network (CNN)***

We implemented the CNN network [12] also using Pytorch. We stacked three one-dimensional convolutional layers following the input layer. The first convolutional layer had an input channel of 21, an output channel of 32, a convolutional kernel size of 5, and a step size of 1. The remaining two layers had an input and output channel size of 32, a convolutional kernel size of 5, and strides of 2. To prevent overfitting, a dropout operation with a rate of 0.3 was implemented after each convolutional layer. After flattening the third convolutional layer, we added a linear layer with 128 neurons, followed by an output layer with 2 neurons. During the training process, focal loss was used as the loss function. The batch size for input data was set to 256. The number of the training epochs was set to 50. The learning rate was set to 0.001. Adam optimizer was used to tune the model parameters.

**References**

1. Breiman LJMl. Random forests 2001;45:5-32.

2. Pedregosa F, Varoquaux G, Gramfort A et al. Scikit-learn: Machine learning in Python 2011;12:2825-2830.

3. Freund Y, Schapire REJJoc, sciences s. A decision-theoretic generalization of on-line learning and an application to boosting 1997;55:119-139.

4. Bao W, Cui Q, Chen B et al. Phage_UniR_LGBM: Phage Virion Proteins Classification with UniRep Features and LightGBM Model. Comput Math Methods Med 2022;2022:9470683. http://doi.org/10.1155/2022/9470683

5. Bao W, Gu Y, Chen B et al. Golgi_DF: Golgi proteins classification with deep forest. Front Neurosci 2023;17:1197824. http://doi.org/10.3389/fnins.2023.1197824

6. Ke G, Meng Q, Finley T et al. Lightgbm: A highly efficient gradient boosting decision tree 2017;30.

7. Hochreiter S, Schmidhuber J. Long short-term memory. Neural Comput 1997;9:1735-1780. http://doi.org/10.1162/neco.1997.9.8.1735

8. Paszke A, Gross S, Massa F et al. Pytorch: An imperative style, high-performance deep learning library 2019;32.

9. Lin T-Y, Goyal P, Girshick R et al. Focal loss for dense object detection. In: Proceedings of the IEEE international conference on computer vision. 2017, p. 2980-2988.

10. Schuster M, Paliwal KK. Bidirectional recurrent neural networks. IEEE Transactions on Signal Processing 1997;45:2673-2681. http://doi.org/10.1109/78.650093

11. Kingma DP, Ba JJapa. Adam: A method for stochastic optimization 2014.

12. Kim YJapa. Convolutional neural networks for sentence classification 2014.
